# Unique characteristics of CpG island methylator phenotype (CIMP) in a Chinese population with colorectal cancer

**DOI:** 10.1186/s12876-019-1086-x

**Published:** 2019-11-05

**Authors:** Jiang Liu, Li Tang, Jinhua Yi, Guimei Li, Youwang Lu, Yu Xu, Shuhua Zhao, Rui Mao, Xiaolu Li, Li Ren, Kunhua Wang

**Affiliations:** 1 Department of Reproduction and Genetics, the First Affiliated Hospital of Kunming Medical University, Kunming, 650032 Yunnan China; 2 Public Technical Service Center, Kunming Institute of Zoology, Chinese Academy of Sciences, Kunming, 650032 Yunnan China; 3 Kunming Biological Diversity Regional Center of Large Apparatus and Equipments, Chinese Academy of Sciences, Kunming, 650032 Yunnan China; 4 Department of Gastrointestinal Surgery, the First Affiliated Hospital of Kunming Medical University, Yunnan Institute of Digestive Disease, Kunming, 650032 Yunnan China; 5 Yunnan Engineering Technology Center of Digestive Disease, Kunming, 650032 Yunnan China; 6 Kunming Engineering Technology Center of Digestive Disease, Kunming, 650032 Yunnan China; 7 School of Stomatology, Kunming Medical University, Kunming, 650500 Yunnan China; 8 Department of Reproductive Gynecology, the First People’s Hospital of Yunnan Province, Kunming, 650031 Yunnan China

**Keywords:** Colorectal cancer, CpG island methylator phenotype, CIMP, *BRAF* mutations, *KRAS* mutations

## Abstract

**Background:**

Molecular characteristics of CpG island methylator phenotype (CIMP) in colorectal cancer (CRC) have been well documented in Western, but not in Chinese, populations.

**Methods:**

We investigated the incidence of CIMP, *BRAF*/*KRAS* mutation, and microsatellite instability (MSI) in a Chinese population with CRC (*n* = 401) and analysed associations between CIMP status and clinicopathological and molecular features.

**Results:**

A total of 41 cases, 310 cases, and 40 cases were classified as CIMP-high, CIMP-low, and CIMP-negative, respectively. We detected a significantly low incidence of *BRAF* mutation in adenomas (2%) and CRC (0.7%), and a relatively low incidence of *KRAS* mutation (24.9%) compared with that in other populations. We also detected a relatively low incidence of CIMP-high (10.2%), which was significantly associated with younger age (≤49 years of age), female sex, and proximal tumour location.

**Conclusions:**

This study revealed unique characteristics of CIMP in a Chinese population with colorectal cancer. Developing specific CIMP markers based on unique populations or ethnic groups will further help to fully elucidate CIMP pathogenesis.

## Background

Based on global estimates in 2012, colorectal cancer (CRC) is the third most commonly diagnosed cancer in males and the second in females, with an estimated 1.4 million cases and 693,900 deaths occurring annually [1]. CRC is currently considered to represent a constellation of heterogeneous subtypes that result from different combinations of genetic events and epigenetic alterations. A series of studies have shown the ability to classify CRC subtypes based on combinations of microsatellite instability (MSI), CpG island methylator phenotype (CIMP), somatic *BRAF* mutation, and/or somatic *KRAS* mutation status [2–11]. Over the years, significant advances have been made in characterizing the molecular genetics and epigenetics of colorectal tumourigenesis, leading to the bench-to-bedside application of biomarkers such as *KRAS*, *BRAF,* and CIMP for personalised medicine [12].

CIMP characterises a subset of CRCs exhibiting a very high frequency of aberrant DNA hypermethylation at “type C” loci, which are defined as loci methylated in cancer, but not in normal, tissues [13]. The CIMP trait has been found to be associated with a variety of clinical, histopathological, and epidemiological characteristics, such as older age, female sex, proximal tumour location, poorly differentiated or mucinous histology, and high rates of MSI and *BRAF* mutation [4, 6, 9, 10, 14–17]. Although there are conflicting data regarding whether CIMP-positive patients receive benefit from adjuvant 5-fluorouracil therapy [4, 9, 18, 19], CIMP status has been evaluated as a predictive marker for chemotherapy responsiveness. Possible explanations for this inconsistency include the use of small case-control studies, differences in the loci used to define CIMP, and different hypermethylation assays used.

Although accumulating evidence indicates that these molecular characteristics (including MSI, CIMP, and *BRAF*/*KRAS* mutation status) have diagnostic, therapeutic, and prognostic significance in CRC [20–22], their incidences, especially those of MSI, *BRAF* mutation, and CIMP, vary considerably among different ethnic groups [8, 22, 23]. In a population-based cohort study, Carethers et al. found that the frequency of MSI among an African-American cohort with colon cancer was half that of a Caucasian cohort, suggesting that once an African-American is diagnosed with colon cancer, the improved survival associated with MSI cancers is more limited in this population [24]. Worldwide, the reported frequency of *BRAF* mutation in different populations varies widely, from 1.1% in Taiwan to 19.8% in the Netherlands [5, 22, 25–30]. Compared with Western populations, a lower frequency of *BRAF* mutation has been observed in most Asian populations, from 1.1% in Taiwan to 7% in North China [5, 25, 26, 28–30]. Similarly, the reported incidence of CIMP in different populations varies widely, from 5.1% in Saudi Arabia to 30% in the United States [2, 6–8, 10, 11, 31–35]. These differences may be related to differences in the methodology and CIMP marker panel used to determine CIMP status in these studies [33]. In addition, studies focusing on the molecular characteristics of CIMP in Chinese populations are very limited. The only relevant study reports a CIMP incidence of 13.12% in a Northeast Chinese population [7]; however, it has been suggested that the CIMP markers used in this study (including *MINT1*, *MINT31*, *p16*, *MLH1*, *MGMT*, *APC*, and *RUNX3*) are not very sensitive and specific for CIMP diagnosis [31].

Due to the lack of data on the molecular characteristics of CRC (including MSI, CIMP, and *BRAF/KRAS* mutation status) in Chinese populations, we sought to utilise a population-based CRC cohort to more accurately determine the prevalence and characteristics of these features in a Chinese CRC population. We comprehensively investigated the incidence of MSI, CIMP, and *BRAF* and *KRAS* mutations in a unique ethnic Chinese CRC population and analysed associations between CIMP status and clinicopathological and molecular features. We also aimed to elucidate the aetiological factors and pathogenesis of CIMP-high CRC in this unique ethnic Chinese population of CRC patients.

## Methods

### Tissue samples

Formalin-fixed, paraffin-embedded archival tissues from 317 CRC patients were retrieved from the Department of Pathology, the First Affiliated Hospital of Kunming Medical University (Kunming, China). Fresh colorectal tumour and surrounding normal tissues were collected at surgery from 84 patients, and representative sections for research were removed by a pathologist. These patients had undergone curative surgery at the First Affiliated Hospital of Kunming Medical University between 2014 and 2016. Patients gave a written informed consent for the use of their bowel tissue for research. Selection was based solely on the availability of archival tissue blocks for the study, and we did not exclude patients with a family history of CRC. Clinicopathological information, including age, sex, tumour location, and tumour stage, was obtained from all 401 patients (Table 1). The cecum, ascending colon, hepatic flexure, transverse colon, and splenic flexure were classified as proximal, while the descending colon, sigmoid colon, and rectum were classified as distal. Tumours were staged on the basis of the pathological tumour-node-metastasis (pTNM) staging system of the American Joint Committee on Cancer (AJCC). The study was approved by the First Affiliated Hospital of Kunming Medical University Ethics Committee.

**Table 1:** Sample informations used in this study

	Normal Tissues (*n* = 84)	Adenoma (*n* = 98)		Colorectal Cancer Tissues			
			StageI (*n* = 77)	StageII (*n* = 161)	StageIII (*n* = 148)	StageIV (*n* = 15)	Total (n = 401)
Male, n (%)	48 (57%)	58 (59%)	40 (52%)	93 (58%)	85 (57%)	10 (67%)	228 (57%)
Female, n (%)	36 (43%)	40 (41%)	37 (48%)	68 (42%)	63 (43%)	5 (33%)	173 (43%)
Age, median (range)	57 (24–81)	59 (20–86)	55.18 (24–81)	54.13 (28–80)	53.91 (20–87)	64.4 (26–85)	54.64 (20–87)
Colon, n (%)	42 (50%)	69 (70%)	31 (40%)	95 (59%)	76 (51%)	12 (80%)	214 (53%)
Rectum, n (%)	42 (50%)	29 (30%)	46 (60%)	66 (41%)	72 (49%)	3 (20%)	187 (47%)

### DNA extraction and bisulphite modification

Through light microscopic examination, we marked tumour areas where tumour cells accounted for 50% or more of all cells and analysed the main histology and differentiation of the tumour. Eight serial 10-μm-thick histological slides of formalin-fixed tumour tissue blocks were used for manual microdissection. Genomic DNA was extracted using the QIAamp DNA FFPE Tissue kit (QIAGEN, Germantown, MD, USA) for formalin-fixed, paraffin-embedded archival tissues and the QIAamp DNA Mini Kit (QIAGEN) for fresh tissues. Bisulphite modification was carried out using an EpiTect Fast DNA Bisulphite Kit (QIAGEN) according to the manufacturer’s instructions.

### DNA methylation analysis

DNA methylation analyses were performed using MethyLight, as previously described [31, 32]. The oligonucleotide sequences of the primers and probes have been described previously [31, 32, 36]. The PCR conditions were as follows: initial denaturation at 95 °C for 10 min, followed by 40 cycles of 95 °C for 15 s and 60 °C for 1 min. M.SssI-treated genomic DNA was used as a completely methylated reference sample to determine the percentage of fully methylated alleles [percentage of methylated reference (PMR)] at a particular locus. The PMR value was calculated by dividing the GENE/ALU ratio of a sample by the GENE/ALU ratio of the M.SssI-treated human genomic DNA sample and multiplying by 100. A PMR cut-off of 4 was used to distinguish methylation-positive (PMR > 4) from methylation-negative (PMR ≤ 4) samples.

Although several marker panels have been proposed to standardise the classification of CIMP-positive [31, 32, 36, 37], we quantified DNA methylation in eight CIMP markers (*CACNA1G*, *CDKN2A*, *CRABP1*, *IGF2*, *MLH1*, *NEUROG1*, *RUNX3*, and *SOCS1*), as these have been shown to be sensitive and specific for CIMP diagnosis [32]. These eight CIMP markers can be divided into three marker panels: CIMP-1 (*CACNA1G*, *CDKN2A*, *CRABP1*, *MLH1*, and *NEUROG1*), described by Ogino and colleagues [36]; CIMP-2 (*CACNA1G*, *IGF2*, *NEUROG1*, *RUNX3*, and *SOCS1*), described by Weisenberger and colleagues [31]; and CIMP-3, including all eight of the markers [32, 33, 38]. For the CIMP-1 panel, CRC cases were considered CIMP-positive if at least four of the markers were methylated [36]; for the CIMP-2 panel, CRC cases were considered CIMP-positive if at least three of the markers were methylated [31]. For the CIMP-3 panel, a cut-off of ≥5/8 methylated markers was used to classify cases into CIMP-high CRC, as usage of this cut-off has shown stronger associations with known clinicopathological or molecular features of CIMP-high CRC in Korea [33]. A cut-off of 1–4/8 methylated markers was used to classify cases into CIMP-low, while a cut-off of 0/8 methylated markers was used to classify cases into CIMP-negative. We evaluated the performance of these three marker panels by comparing their associations with clinicopathological features of CRC that have been previously reported to be associated with CIMP-positive status, including older age, female sex, proximal location, *BRAF* mutation, and MSI status.

### Mutational analysis of *KRAS* codons 12 and 13 and *BRAF* codon 600

Tumour DNA was tested for the *BRAF* codon 597 and 600 mutations and *KRAS* codons 12 and 13 mutations in 98 adenomas and 401 CRC samples. Mutations of *BRAF* (nucleotides 1790 and 1799) and *KRAS* (nucleotides 35 and 38) were analysed by genotyping assay on the MassARRAY platform (Sequenom, San Diego, CA, USA). PCR and extension primers for these mutations were designed using MassARRAY Assay Design 3.0 software (Sequenom) and applying default single-base extension settings and default parameters (Additional file 1: Table S1). DNA was amplified by PCR, and a single-base extension reaction was performed using a custom mixture of nucleotides and extension primers that hybridised immediately adjacent to the mutations. Reaction products were transferred to a SpectroCHIP (Sequenom), and mass differences were analysed using MALDI-TOF mass spectrometry to identify the extended base at the possible mutation site. Repeat Sanger sequencing on an ABI PRISM 3730 Genetic Analyzer (Applied Biosystems, Foster City, CA, USA) was used to reconfirm the results of MassARRAY and rule out the possibility that any mutations were missed due to the sensitivity of the MassARRAY platform. Primers used to amplify and sequence exon 15 of *BRAF* and exon 1 of *KRAS* are shown in Additional file 1: Table S1.

### MSI analysis

For determination of MSI status, we used a panel of 5 microsatellite markers (*BAT25*, *BAT26*, *NR-21*, *NR-24*, and *MONO-27*) to classify fresh tumour tissues as MSI-high (MSI-H), MSI-low (MSI-L), or microsatellite stable (MSS). MSI-H was defined as ≥2 markers demonstrating novel alleles compared to non-tumour tissues, MSI-L was defined as 1 marker with a novel allele, and MSS was defined as no markers with novel alleles.

### Statistical analysis

For statistical analysis, the χ^2^ test or Fisher’s exact test (for categories with *n* < 10) was performed on categorical data using the IBM SPSS Statistics 22.0 software. All *P* values were two-sided, and statistical significance was set at *P* ≤ 0.05.

## Results

### Clinicopathological characteristics

Of the 401 patients, the proportion of males (57%) was slightly higher than that of females (43%), with a male to female ratio of 1.32:1. Patient age at presentation ranged from 20 to 87 years (median, 54.64 years). There were 309 patients (77.1%) that presented with stage II or III disease, while 77 patients (19.2%) were diagnosed with stage I disease and 15 patients (3.7%) were diagnosed with stage IV disease. There were 214 patients (53.4%) whose primary tumours were derived from the colon, while the tumours in the remaining 187 patients (46.6%) were derived from the rectum. Detailed sample information is summarised in Table 1.

### *BRAF* and *KRAS* mutations

Of the 401 CRC specimens analysed for *BRAF* and *KRAS* mutations using the MassARRAY platform, *BRAF* mutation was observed in three cases, with an incidence of 0.7% (3/401); *KRAS* mutation was detected in 100 cases, with an incidence of 24.9% (100/401) (Table 2). For the 98 adenoma samples, *BRAF* mutation was observed in two cases, with an incidence of 2% (2/98), and *KRAS* mutation was detected in 23 cases, with an incidence of 23.5% (23/98). All five *BRAF* mutations were V600E mutations, while *KRAS*-mutated cases showed mutations at codon 12 (67%) and codon 13 (33%). Repeat Sanger sequencing was conducted for 229 specimens, including all of the *BRAF*- and *KRAS*-mutated cases. The results of repeat Sanger sequencing were in accordance with MassARRAY analyses, with no new mutations identified.

**Table 2:** Clinicopathologic and molecular characteristics of CRCs

Demographics	CIMP-1 (≥4/5)			CIMP-2 (≥3/5)			CIMP-3 (≥5/8)			
	Positive (8.5%)	Negative	*P*	Positive (12%)	Negative	*P*	High (10.2%)	Low (77.3%)	Negative (12.5%)	*P*
Age			0.1871			0.1108				**0.0296**
≤49	15 (3.7%)	108 (26.9%)		22 (5.5%)	101 (25.2%)		20 (5%)	86 (21.4%)	17 (4.2%)	
50–59	11 (2.7%)	135 (33.7%)		14 (3.5%)	132 (32.9%)		11 (2.7%)	120 (29.9%)	15 (3.7%)	
≥60	8 (2%)	124 (30.9%)		12 (3%)	120 (29.9%)		10 (2.5%)	104 (25.9%)	18 (4.5%)	
Gender			**0.0046**			**0.0063**				**0.0011**
Men	11 (2.7%)	217 (54.1%)		18 (4.5%)	210 (52.4%)		13 (3.2%)	186 (46.4%)	29 (7.2%)	
Women	23 (5.7%)	150 (37.4%)		30 (7.5%)	143 (35.7%)		28 (7%)	124 (30.9%)	21 (5.2%)	
Tumor location			**0.0142**			0.0552				**0.0065**
Distal	19 (4.7%)	281 (70.1%)		30 (7.5%)	270 (67.3%)		23 (5.7%)	244 (60.8%)	33 (8.2%)	
Proximal	15 (3.7%)	86 (21.4%)		18 (4.5%)	83 (20.7%)		18 (4.5%)	66 (16.5%)	17 (4.2%)	
Stage			0.1697			0.5059				0.5459
I	5 (1.2%)	72 (18.0%)		9 (2.2%)	68 (17%)		6 (1.5%)	61 (15.2%)	10 (2.5%)	
II	17 (4.2%)	144 (35.9%)		24 (6%)	137 (34.2%)		21 (5.2%)	119 (29.7%)	21 (5.2%)	
III	9 (2.2%)	139 (34.7%)		14 (3.5%)	134 (33.4%)		13 (3.2%)	120 (29.9%)	15 (3.7%)	
IV	3 (0.7%)	12 (3.0%)		1 (0.2%)	14 (3.5%)		1 (0.2%)	10 (2.5%)	4 (1%)	
*BRAF* status			0.2339			**0.039**				0.2771
Wild type	33 (8.2%)	365 (91.0%)		46 (11.5%)	352 (87.8%)		40 (10%)	308 (76.8%)	50 (12.5%)	
Mutation	1 (0.2%)	2 (0.5%)		2 (0.5%)	1 (0.2%)		1 (0.2%)	2 (0.5%)	0 (0)	
*KRAS* status			0.0957			0.1072				0.1033
Wild type	21 (5.2%)	280 (69.8%)		31 (7.7%)	270 (67.3%)		26 (6.5%)	234 (58.4%)	41 (10.2%)	
Mutation	13 (3.2%)	87 (21.7%)		17 (4.2%)	83 (20.7%)		15 (3.7%)	76 (19%)	9 (2.2%)	
MSI status			1			0.287				0.7373
MSS	5 (6.1%)	53 (64.6%)		10 (12.2%)	48 (58.5%)		8 (9.8%)	44 (53.7%)	6 (7.3%)	
MSI-low	1 (1.2%)	18 (22.0%)		2 (2.4%)	17 (20.7%)		2 (2.4%)	15 (18.3%)	2 (2.4%)	
MSI-high	0 (0)	5 (6.1%)		2 (2.4%)	3 (3.7%)		1 (1.2%)	4 (4.9%)	0 (0)	

Bold *P* value indicates *P* ≤ 0.05

### MSI analysis

MSI status was determined in 82 CRC patients due to inadequate DNA, lack of paired normal tissues, or technical issues with the remaining specimens. The incidences of MSI-H, MSI-L, and MSS were 6.1% (5/82), 23.2% (19/82), and 70.7% (58/82), respectively (Table 2).

### CIMP prevalence and correlations with clinicopathological and molecular characteristics

**Fig. 1:**
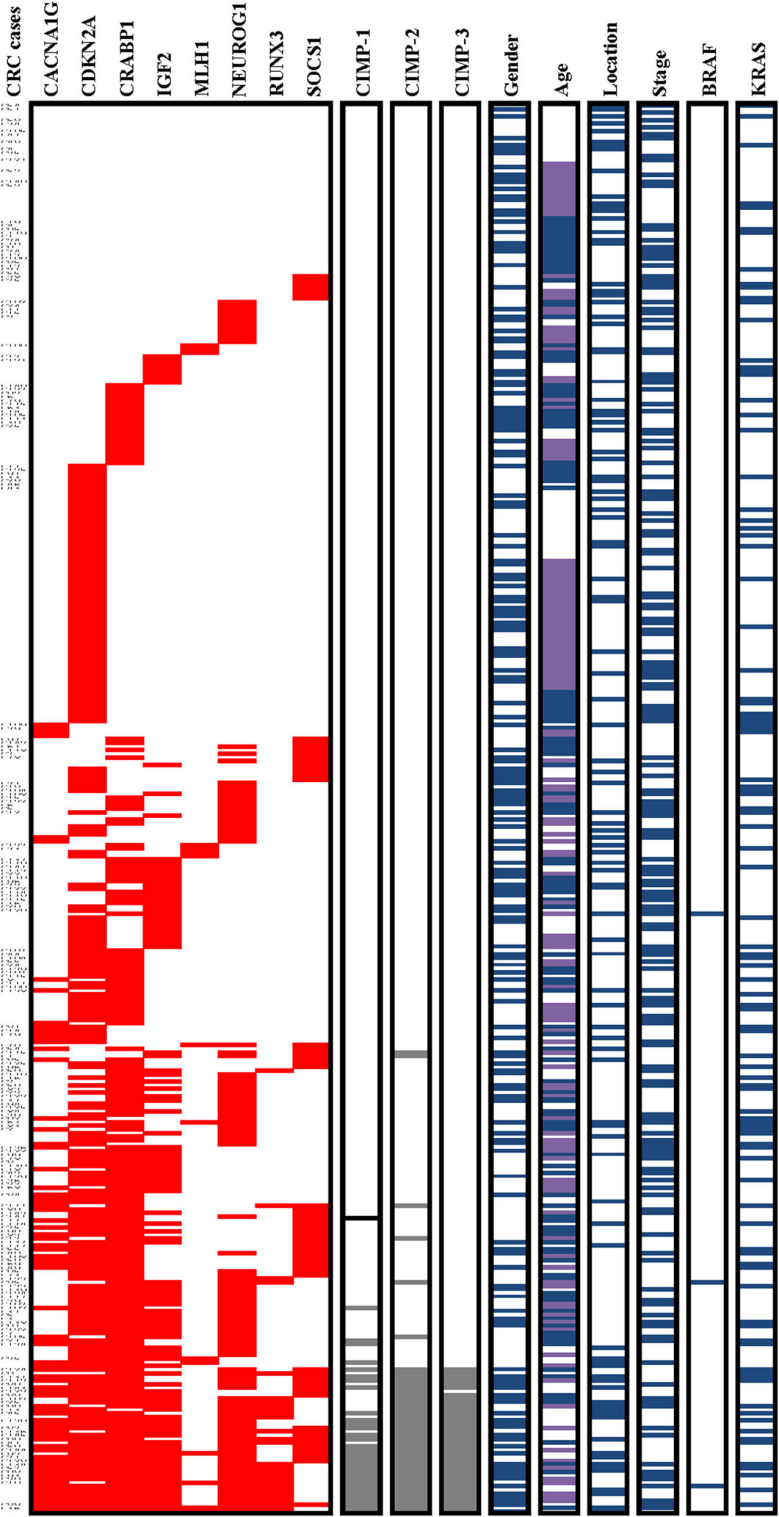
Comparative analysis of CIMP marker panel performance. Red bars represent methylation-positive CIMP markers, and grey bars represent CIMP-positive (CIMP-1 and CIMP-2) or CIMP-high (CIMP-3) classifications using three differently defined CIMP panels. Blue bars represent female sex, older age (≥60 years), proximal colon location, higher stage (III, IV), *KRAS* mutation, and *BRAF* mutation. Purple bars represent ages 50–59

We obtained 401 colorectal cancer specimens and successfully quantified DNA methylation in eight CIMP-specific gene promoters (*CACNA1G*, *CDKN2A*, *CRABP1*, *IGF2*, *MLH1*, *NEUROG1*, *RUNX3*, and *SOCS1*) using MethyLight technology. Methylation frequencies were 20% for *CACNA1G* (80 cases), 57.6% for *CDKN2A* (231 cases), 49.6% for *CRABP1* (199 cases), 30.7% for *IGF2* (123 cases), 3.7% for *MLH1* (15 cases), 31.2% for *NEUROG1* (125 cases), 7.2% for *RUNX3* (29 cases), and 17% for *SOCS1* (68 cases). A summary of the clinicopathological and molecular characteristics of CRC cases according to each of the three CIMP panels (CIMP-1, CIMP-2, and CIMP-3) is provided in Fig. 1 and Table 2. CIMP-positive cancers were identified in 34 cases (8.5%) using the CIMP-1 panel and 48 cases (12%) using the CIMP-2 panel. For the CIMP-3 marker panel, 41 cases (10.2%), 310 cases (77.3%), and 50 cases (12.5%) were classified as CIMP-high, CIMP-low, and CIMP-negative, respectively. The frequency of CIMP-1-positive cases was significantly higher in women (13.3%) than in men (4.8%, *P* < 0.01) and significantly higher in cases with proximal tumour locations (14.9%) than in those with distal tumour locations (6.3%, *P* < 0.05). CIMP-2 positivity was significantly more frequent in women (17.3%) than in men (7.9%, *P* < 0.001) and was associated with a *BRAF* mutant type (66.7%) rather than *BRAF* wild type (11.6%, *P* < 0.05). For the CIMP-3 panel, of particular note is the fact that the frequency of CIMP-high varied by age, with a significantly higher rate in patients ≤49 years of age (16.3%) compared to that in patients 50–59 years of age (7.5%) and ≥ 60 years of age (7.6%, *P* < 0.05). Moreover, CIMP-high was significantly more frequent in women (16.2%) than in men (5.7%, *P* < 0.01) and in proximal tumour locations (17.8%) than in distal tumour locations (7.7%, *P* < 0.01). No significant differences were observed in other clinicopathological characteristics among the CIMP phenotypes for the three panels of CIMP markers. Because previous studies suggested that the CIMP-3 panel outperformed the CIMP-1 and CIMP-2 panels both in Western CRC populations and in Asian CRC populations [32, 33], we used the CIMP-3 panel for the determination of CIMP in this study.

### Assessment of individual CIMP methylation markers

To compare the performance of the eight individual methylation markers for the determination of panel-specific CIMP status, the sensitivity and specificity were calculated for each of the eight markers among all 401 tumours (Table 3). As shown in Table 3, *CACNA1G*, *CDKN2A*, *CRABP1*, and *NEUROG1* demonstrated very high sensitivity (≥97%) in determining CIMP-1 status. Similarly, *CRABP1* and *NEUROG1* demonstrated high sensitivity (≥90%) in determining CIMP-2 status, and *CDKN2A*, *CRABP1*, and *NEUROG1* demonstrated very high sensitivity (≥97%) in determining CIMP-3 status. For all three panels, *MLH1* and *RUNX3* exhibited superior specificity (≥96%).

**Table 3:** Sensitivity and specificity of each marker for determination of CIMP-high

Marker	Total no.	CIMP-1 (≥4/5)		CIMP-2 (≥3/5)		CIMP-3 (≥5/8)	
		Positive (sensitivity)^a^	Negative (specificity)^b^	Positive (sensitivity) ^a^	Negative (specificity) ^b^	Positive (sensitivity) ^a^	Negative (specificity) ^b^
	401	34 (8.5%)	367	48 (12%)	353	41 (10.2%)	360
*CACNA1G*							
(+)	80 (20%)	33 (97%)	47	38 (79%)	42	34 (83%)	46
(−)	321	1	320 (87%)	10	311 (88%)	7	314 (87%)
*CDKN2A*							
(+)	231 (57.6%)	33 (97%)	198	38 (79%)	193	37 (90%)	194
(−)	170	1	169 (46%)	10	160 (45%)	4	166 (46%)
*CRABP1*							
(+)	199 (49.6%)	34 (100%)	165	45 (94%)	154	40 (98%)	159
(−)	202	0	202 (55%)	3	199 (56%)	1	201 (56%)
*IGF2*							
(+)	123 (30.7%)	24 (71%)	99	40 (83%)	83	34 (83%)	89
(−)	278	10	268 (73%)	8	270 (77%)	7	271 (75%)
*MLH1*							
(+)	15 (3.7%)	5 (15%)	10	4 (8.3%)	11	4 (9.8%)	11
(−)	386	29	357 (97%)	44	342 (97%)	37	349 (97%)
*NEUROG1*							
(+)	125 (31.2%)	33 (97%)	92	43 (90%)	82	39 (95%)	86
(−)	276	1	275 (74%)	5	271 (77%)	2	274 (76%)
*RUNX3*							
(+)	29 (7.2%)	16 (47%)	13	27 (56%)	2	25 (61%)	4
(−)	372	18	354 (96%)	21	351 (99%)	16	356 (99%)
*SOCS1*							
(+)	68 (17%)	13 (38%)	55	24 (50%)	44	19 (46%)	49
(−)	333	21	312 (85%)	24	309 (88%)	22	311 (86%)

^a^Sensitivity of each marker is defined as the number of CIMP-high cases positive for a given marker divided by the number of all CIMP-high cases

^b^Specificity of each marker is defined as the number of non-CIMP-high cases negative for a given marker divided by the number of all non-CIMP-high cases

## Discussion

Due to accumulating evidence indicating that certain molecular characteristics (including CIMP, *BRAF* mutation, *KRAS* mutation, and MSI status) have diagnostic, therapeutic, and prognostic significance in CRC personalised medicine and incidences that vary considerably among different ethnic or geographic populations, this study determined the frequency of CIMP, *BRAF/KRAS* mutation, and MSI in a unique ethnic Chinese population-based CRC cohort. Surprisingly, we detected a significantly low incidence of *BRAF* mutation, both in adenomas (2%) and in CRC (0.7%), and a relatively low incidence of *KRAS* mutation (24.9%) compared with that in other populations [9, 10, 29, 32, 33, 39, 40]. We also detected a relatively low incidence of CIMP-high (10.2%) in our CRC population. Of note, CIMP-high was significantly associated with younger age (≤49 years old), female sex, and proximal tumour location, whereas no significant associations were observed with tumour stage, *BRAF* mutation, *KRAS* mutation, or MSI status. In addition, by comparing the accuracy of the associations of the three CIMP marker panels with previously known clinicopathological features of CIMP-positive CRC, our data indicated that the CIMP-3 panel outperformed the CIMP-1 and CIMP-2 panels in most comparisons. Therefore, consistent with analyses in American and South Korean CRC populations [32, 33], CIMP-3 is currently the optimal marker panel for the determination of CIMP status in the Chinese population with CRC.

*BRAF* mutation has been considered a biomarker with diagnostic, therapeutic, and prognostic significance in CRC [41, 42]. In this study, the BRAF-V600E mutation was identified in only 0.7% (3/401) of all CRC cases and 2% (2/98) of adenoma cases. This implies a very limited role of the *BRAF* gene in the pathogenic process of CRC and a much lower clinical significance of *BRAF* mutation in Chinese populations than in Western populations [2, 6, 9, 10]. *BRAF* mutation is tightly associated with MSI-H and *MLH1* methylation in Western CRC populations [31, 42], but the main reason of MSI-H (6.1%, 5/82) and *MLH1* methylation (3.7%, 15/401) in Chinese CRC populations remains unclear due to very low incidences of *BRAF* mutation. Although very low incidences of *BRAF* mutation were observed in Saudi Arabia and Israel, with frequencies of 2.5 and 5%, respectively [8, 43], and lower frequencies have been observed in most Asian populations, ranging from 1.1% in Taiwan [28] to 2.3–7% in China [25, 29, 44], 4.7–6.7% in Japan [5, 30], and 4.1% in South Korea [40], the incidence revealed in this study is the lowest observed thus far compared with previous reports worldwide [8, 10, 42]. In addition, concordant with previous reports that the incidence of *BRAF* mutation varies widely among CRC populations even within the same region or country [8, 45, 46], three previous studies have reported varied incidences of *BRAF* mutation in CRC populations from different areas of China. A *BRAF* mutation frequency of 2.3% (5/220) was observed in Shanghai [44], 4.4% (20/453) was observed in Beijing [29], and 7% (14/200) was observed in Shanxi province [25], with the lowest incidence of 0.7% (3/401) from the population in this study from Yunnan province. Yunnan province has the most ethnic minorities in China: among the 26 nationalities in Yunnan, 15 of them are native ethnic minorities. Therefore, the fact that the lowest incidence of *BRAF* mutation was observed in a Yunnan CRC population may be due to differences in ethnic populations and the associated variation in underlying genetic and epigenetic backgrounds, as well as environmental influences such as food habits, smoking, drinking, and other unknown factors.

The CIMP-1 panel was first developed by Ogino and colleagues [36]; in their study, 17% (78) of the 460 evaluated CRC specimens were classified as CIMP-positive. In our study, CIMP-1-positive cancers were identified in 34 cases (8.5%) among the 401 CRC specimens. CIMP-2 was first developed by Weisenberger and colleagues [31], who reported that 18% (33) of their 187 CRC specimens were classified as CIMP-positive. In our study, CIMP-2-positive cancers were identified in 48 cases (12%) of the 401 CRC specimens. In 2007, the CIMP-3 panel was first proposed by Ogino and colleagues [32]. In their study, 18% (163) of the 920 CRC specimens were classified as CIMP-high. Later, Kim et al. used the same CIMP marker panel and classified 12% (37/320) of South Korean CRC patients as CIMP-high [33]. In our study, CIMP-high cancers were identified in 41 cases (10.2%) of the 401 CRC specimens. In general, based on the same CIMP marker panel and the same CIMP-high criterion, the frequency of CIMP-high cancers in our CRC population was relatively lower than that of the American CRC population but similar to that of the South Korean population. Of note, although two previous studies determined the frequency of CIMP-positive cancers in Chinese CRC populations [7, 47], these did not use the recognised CIMP markers that we used in this study to classify CIMP cancers, so their results are not comparable with ours and others’. Li et al. used *MLH1*, *MGMT*, *p16*, *APC*, *MINT1*, *MINT31*, and *RUNX3* as CIMP panel markers and classified 13.12% (37/282) of patients as CIMP-high [7]. Wang et al. used *p14ARF*, *hMLH1*, *p16INK4a*, *MGMT*, and *MINT1* as CIMP markers and identified 12 CIMP-positive cases (24%) in 50 CRC specimens [47]. Therefore, our results once again show that differences in CIMP marker panels may contribute to discrepancies in CIMP frequency, even for the same CRC population.

Studies on American CRC populations have indicated that CIMP is significantly associated with female sex, older age, proximal tumour location, MSI, *BRAF* mutation, and wild-type *KRAS* [31, 36, 48]. However, in this study, CIMP-high was significantly associated with female sex, younger age, and proximal tumour location. No significant association was observed with other clinicopathological characteristics, including MSI, *BRAF* mutation, or wild-type *KRAS*. Notably, this is the first study to report an association between CIMP-high and younger age. A possible reason for the lack of an association between CIMP-high and MSI may be the limited sample size (*n* = 82) included in the MSI analysis. The lack of an association with *BRAF* mutation may be due to the very low incidence of *BRAF* mutation observed in this population (0.7%). Alternatively, just as accumulating evidence has demonstrated that differences in CIMP marker panels may contribute to discrepancies in CIMP frequency, even in the same CRC population, the same CIMP marker panel may not be suitable for the diagnosis of CIMP among different populations. For example, using the same CIMP-3 marker panel with the same CIMP-high criterion, Ogino and colleagues found that *CRABP1*, *IGF2*, and *NEUROG1* demonstrated very good sensitivity (≥95%), whereas *CACNA1G*, *MLH1*, *RUNX3*, and *SOCS1* showed superior specificity (≥90%) in 920 American CRC cases [32]. However, in this study, we found that *CDKN2A*, *CRABP1*, and *NEUROG1* demonstrated very good sensitivity (≥97%), while *MLH1* and *RUNX3* exhibited superior specificity (≥96%) when using CIMP-3. In contrast, the sensitivity of *IGF2* was 83%, and the specificities of *CACNA1G* and *SOCS1* were 87 and 86%, respectively, in our CRC population, while the sensitivity of *CDKN2A* was 87% among American CRCs [32]. Thus, the same CIMP markers exhibit different performances for the determination of CIMP in different CRC populations. While all of the eight CIMP markers in the CIMP-3 marker panel were developed based on American CRC populations [31, 32, 36], increasing evidence has shown that the incidence of CIMP varies widely among different populations [41] or ethnic backgrounds [48]. Therefore, as with the low *BRAF* mutation frequency detected in this study, we speculate that different ethnic populations with different underlying genetic and epigenetic backgrounds and environmental influences, such as food habits, lifestyle habits, and environmental exposures, may contribute to the varied CIMP characteristics and prevalence observed. The development of specific CIMP markers based on unique CRC populations or ethnicities will further help to fully elucidate the pathogenesis of CIMP.

CIMP-positive tumours are generally thought to develop through the serrated neoplasia pathway and are associated with *BRAF* mutation [31, 49]. Furthermore, the frequency of *BRAF* mutation is much higher in serrated adenomas than in conventional adenomas. For example, the frequency of *BRAF* mutation was found to be 67% among 200 traditional serrated adenomas, but no *BRAF* mutations were identified in 50 control tubulovillous adenomas [50]. However, in this study, although the incidence of CIMP-high was 10.2%, *BRAF* mutation was very rare among CRC cases (only 0.7%). Similarly, the *BRAF* mutation frequency was only 2% in adenomas. Therefore, as the acknowledged precursors of CIMP-positive CRC, the contributions of advanced serrated adenomas to the incidence of CIMP-high CRC should be very limited in our CRC population. We believe that the unique ethnic population and the associated underlying genetic and epigenetic backgrounds may contribute to the unique molecular characteristics of CIMP-high CRC in our population. Associations between CIMP-positive CRC and environmental exposures have been carefully investigated, and associations of smoking and obesity with CIMP-positive CRC were evident only for females in an American population [48]. Therefore, characterising CIMP-high CRC using genome-scale technologies and dissecting the separate aetiological factors associated with smoking, alcohol use, obesity, and physical inactivity will further elucidate the pathogenesis of CIMP-high CRC for this unique ethnic population.

## Conclusions

This study detected a significantly low incidence of *BRAF* mutation in adenomas (2%) and CRC (0.7%), and a relatively low incidence of *KRAS* mutation (24.9%) compared with that in other populations. We also detected a relatively low incidence of CIMP-high (10.2%), which was significantly associated with younger age (≤49 years of age), female sex, and proximal tumour location. To our knowledge, this is the first study to suggest an association between CIMP-high and younger age, while most previous studies have associated CIMP-high and older age. We speculate that different genetic backgrounds and lifestyle habits may contribute to the unique pathogenesis of CIMP-high CRC among the ethnic Chinese population. Developing specific CIMP markers based on unique populations or ethnic groups will further help to fully elucidate CIMP pathogenesis.

## Supplementary information

**Additional file 1: Table S1.** *KRAS* and *BRAF* primers used for MassARRAY analyses and Sanger sequencing in this study.

## Data Availability

The datasets generated and analyzed during the current study are available from the corresponding author on reasonable request.

## Abbreviations

AJCC: American Joint Committee on Cancer
CIMP: CpG island methylator phenotype
CRC: Colorectal cancer
MSI: Microsatellite instability
MSI-H: MSI-high
MSI-L: MSI-low
MSS: Microsatellite stable
PMR: Percentage of methylated reference
pTNM: Pathological tumour-node-metastasis
