# Supplementary material for: Unique characteristics of CpG island methylator phenotype (CIMP) in a Chinese population with colorectal cancer
Source: BMC Gastroenterol. 2019 Nov 5;19:173. doi: 10.1186/s12876-019-1086-x (PMC6833289; doi:10.1186/s12876-019-1086-x)
Supplement: Supplementary file 1 — Additional file 1: Table S1. KRAS and BRAF primers used for MassARRAY analyses and Sanger sequencing in this study. [file 12876_2019_1086_MOESM1_ESM.docx]

Table **S**1

KRAS and BRAF primers used for MassARRAY analyses and Sanger sequencing in this study

| Primer Name | Forward Primer Sequence | Reverse Primer Sequence | Extended Primer Sequence |
| --- | --- | --- | --- |
| KRAS_35_rs727503108_Mass | ACGTTGGATGAGGCCTGCTGAAAATGACTG | ACGTTGGATGGCTGTATCGTCAAGGCACTC | CACTCTTGCCTACGCCA |
| KRAS_38_rs112445441_Mass | ACGTTGGATGGCTGTATCGTCAAGGCACTC | ACGTTGGATGAGGCCTGCTGAAAATGACTG | TGTGGTAGTTGGAGCTGGTG |
| KRAS_exon 1_Sanger | GTTTGTATTAAAAGGTACTGGTGGA | ATAAGTACTCATGAAAATGGTCAGAG |  |
| BRAF_1799_rs113488022_Mass | ACGTTGGATGTCTTCATGAAGACCTCACAG | ACGTTGGATGTTCAAACTGATGGGACCCAC | CCCACTCCATCGAGATTTC |
| BRAF_1790_rs121913366_Mass | ACGTTGGATGTCCTTTACTTACTACACCTC | ACGTTGGATGCCACTCCATCGAGATTTCAC | GTAAAAATAGGTGATTTTGGTC |
| BRAF_exon 15_Sanger | CCTAAACTCTTCATAATGCTTGCTC | CCTTCAATGACTTTCTAGTAACTCAG |  |
